# Socio-demographic determinants of skilled birth attendant at delivery in rural southern Ghana

**DOI:** 10.1186/s13104-017-2591-z

**Published:** 2017-07-11

**Authors:** Alfred Kwesi Manyeh, David Etsey Akpakli, Vida Kukula, Rosemond Akepene Ekey, Solomon Narh-Bana, Alexander Adjei, Margaret Gyapong

**Affiliations:** 1 Dodowa Health Research Centre, P. O. Box. DD1, Dodowa, Accra, Ghana; 2 Shai-Osudoku District Hospital, Dodowa, Ghana; 3 Ghana Health Services, Accra, Ghana

**Keywords:** Skilled attendants, Antenatal care, Pregnancy, Determinant, Dodowa, Ghana

## Abstract

**Background:**

Maternal mortality is the subject of the United Nations’ fifth Millennium Development Goal, which is to reduce the maternal mortality ratio by three quarters from 1990 to 2015. The giant strides made by western countries in dropping of their maternal mortality ratio were due to the recognition given to skilled attendants at delivery. In Ghana, nine in ten mothers receive antenatal care from a health professional whereas only 59 and 68% of deliveries are assisted by skilled personnel in 2008 and 2010 respectively. This study therefore examines the determinants of skilled birth attendant at delivery in rural southern Ghana.

**Methods:**

This study comprises of 1874 women of reproductive age who had given birth 2 years prior to the study whose information were extracted from the Dodowa Health and Demographic Surveillance System. The univariable and multivariable associations between exposure variables (risk factors) and skilled birth attendant at delivery were explored using logistic regression.

**Results:**

Out of a total of 1874 study participants, 98.29% of them receive antenatal care services during pregnancy and only 68.89% were assisted by skilled person at their last delivery prior to the survey. The result shows a remarkable influence of maternal age, level of education, parity, socioeconomic status and antenatal care attendance on skilled attendants at delivery.

**Conclusion:**

Although 69% of women in the study had skilled birth attendants at delivery, women from poorest households, higher parity, uneducated, and not attending antenatal care and younger women were more likely to deliver without a skilled birth attendants at delivery. Future intervention in the study area to bridge the gap between the poor and least poor women, improve maternal health and promote the use of skilled birth at delivery is recommended.

## Background

Annually, more than 200 million women become pregnant globally and 40% of them are estimated to experience pregnancy-related health problems with 15% experiencing serious or long-term complications [1]. This is underlined by the continuing occurrence of more than half a million deaths of women yearly due to pregnancy-related complications in the world, of which 99% are distributed by developing countries [2]. Lifetime risk of death due to pregnancy-related complications is 250-fold higher among women in developing countries in developed countries and it has been estimated that 88–98% of these deaths are avoidable [1].

The Millennium Development Goal (MDG) on maternal health is to reduce maternal mortality by three quarters between 1990 and 2015. One of the health care indicators identified to monitor the “process” of reducing maternal mortality is the proportion of births attended by skilled health personnel. A study revealed that the giant strides made by Western countries in dropping of their maternal mortality figures was due to much recognition given to skilled attendants at delivery in their countries [3].

While a number of studies [4–11] found that majority of women in developing countries utilize antenatal care (ANC) during their pregnancy, their deliveries often lack skilled birth attendants. The importance of skilled attendance at delivery has been recognised by a number of studies [4, 9, 12–24], and it is crucial for decreasing maternal and neonatal mortality, yet many women in low and middle income countries deliver outside of health facilities without skilled attendance at childbirth [3]. ANC visits serve to encourage women to have skilled attendants at birth at a facility, who can provide life-saving emergency obstetric care (EOC) interventions to women who develop serious complications.

In Ghana, many women do not seek skilled health care due to cost of service, the distance to the health facility, and quality of care thereby bringing about a low coverage of 59% skilled deliveries despite the various strategies being put in place. The 2008 Ghana Demographic Health Survey (GDHS) report shows that, over nine in ten mothers (95%) receive antenatal care from a health professional however only 59% of deliveries were assisted by skilled personnel [25].

Trend of skilled delivery services assessed in the Ga East Municipal area of Greater Accra Region of Ghana revealed that although antenatal services is at an appreciable level of 67% in 2010, skilled deliveries however is as low as 37.5%. This depicts a marginal increase of about 6% over the previous year‘s coverage which in 2010 was below the national and global targets of 60 and 85% respectively [26].

In 2003, Ghana introduced the free maternal delivery services to solve the cost of services. With prior establishment of the Community-based Health Planning and Services (CHPS) initiative to increase access, health education on benefits of utilization of maternity services and other activities towards improving maternal health care in the country. Such strategies were expected to show a corresponding increase in the coverage of skilled deliveries but this however, was not the case [27]. It is clear that, improving maternal health remains the most elusive of the MDG in Ghana. The only way this can be improved is if women receive supervised care from health professionals. The aim of this study is to examine factors contributing to this low trend of skilled deliveries in the Dodowa Health and Demographic Surveillance System.

## Methods

### Study area

Data for this study were extracted from Dodowa Health and Demographic Surveillance System (DHDSS) site database. The DHDSS is located in the south-eastern part of Ghana and operates within the boundaries of the Shai-Osudoku and Ningo-Prampram districts [28]. The DHDSS site lies between latitude 5°45′ south and 6°05′ north and longitude 0°05′ east and 0°20′ west with a land area of 1528.9 sq km. It is about 41 km from the national capital, Accra [28, 29]. The two districts cover a population of 115,754 people in 380 communities in 23,647 households covering a total land area of 1442 sq km. The inhabitants are predominantly subsistence farmers, fishermen and petty traders [29]. Road networks in the DHDSS are usually inaccessible during the wet seasons, making access to health and other services a challenge.

The DHDSS visits every household in the demographic surveillance area twice in a year to collect data on demographic, migration and other health indicators [29]. Health care service in the DHDSS is delivered by hospitals, health centres, CHPS zones, private facilities, clinics, maternity homes, mission clinics and quasi government clinics.

### Study population

The study population comprised women of reproductive age (15–49 years) who were resident in the DHDSS from 1st January 2011 to 31st December 2011 who had given birth not more than 1 year prior to the study.

### Outcome and exposure variables

The outcome variable for this study is skilled birth attendant (SBA) at delivery which is binary recorded as: 1 “Skilled person” and 0 “No Skilled person”. From the questionnaire and data available, we selected 8 exposure variables which were based on available literature and has the potential to influence the place of delivery. These exposure variables includes: maternal age, education, parity, first live birth or not, marital status, ANC attendance, and wealth index.

The wealth index is a proxy measure of a household’s long term standard of living; it’s based on social status, assets ownership, and availability of utilities, among others. The index measures were combined into a wealth index, using weights derived through principal component analysis (PCA) [30]. The proxies from the PCA were divided into five quintiles; poorest, very poor, poor, less poor and least poor. Maternal ages at delivery were calculated using the mothers’ and babies’ birthdates.

### Statistical methods

To ensure the assumption of independence of observations, an initial assessment of clustering at household level was carried out since women from the same household may have same or similar health seeking behaviours. The assessment shows that the assumption of independence was upheld.

The univariable associations between each exposure variable (risk factor) and SBA were explored, and those significant at P < 0.05 were entered together into a multiple logistic regression model. Collinearity between all variables and models fitted with and without adjustment was checked using Pearson’s correlation matrix. All analyses were conducted in Stata version 12 and results were presented in the form of tables and summary statistics.

## Results

### Background characteristics

Table 1 presents the background characteristics of the research participants. The mean age of the participants (n = 1874) was 27.41 ± 6.87 years, with ages ranging from 15 to 49. Large proportion of the study participants (73.11%) were of the Ga-Dangme ethnic group. Christians account for the highest proportion in religious affiliation (92.16%) followed by Islamic (5.34%) and the remaining religious denominations contributed only small proportions of the participants. Majority (27.21%) of the respondents were petty traders while 25.45 and 15.96% were unemployed and farmers respectively and the remaining categories of occupation contributed smaller proportion of participants. While 34.47% of the study participants had junior high/middle school level of education, 30.26 and 27.32% had primary and no education respectively. It is well noting that only small proportion (7.95%) of the participants had senior high and above level of education. While 48.40% of the women had their marital status as cohabiting, 29.35 and 18.62% were single (never marry) and married respectively. Majority of the respondents (28.98%) had parity of 1, while 24.76, 17.56 and 16.81% had parity 2, 3, and 5+ respectively. Of a sample of 1873, 1291 (68.89%) were assisted by skilled person at their last delivery prior to the survey. The results show that 98.29% of the women receive ANC services during pregnancy and 71.02% had previous live birth.

**Table 1:** Socio-demographic characteristics of study population (women aged 15–49)

Characteristics	Frequency^a^	Proportion (%)
Age group		
15–19	223	11.9
20–24	494	26.36
25–29	498	26.57
30–34	348	18.57
35–39	207	11.05
40+	104	5.55
Mean = 27.41 (SD = 6.87)		
Ethnicity		
Ga-Dangme	1370	73.11
Akan	101	5.39
Ewe	298	15.9
Northern	97	5.18
Others	8	0.43
Religion		
Christianity	1727	92.16
Islamic	100	5.34
Traditional	22	1.17
Others	25	1.33
Occupation		
Unemployed	477	25.45
Farmer	299	15.96
Artisan	252	13.45
Petty trader	510	27.21
Civil servant	30	1.6
Student	265	14.14
Others	41	2.19
Level of education		
No education	512	27.32
Primary	567	30.26
Junior high/middle school	646	34.47
Senior high and above	149	7.95
Marital status		
Single	550	29.35
Married	349	18.62
Separated/divorced	32	1.71
Cohabiting	907	48.4
Widowed	9	0.48
Missing	27	1.44
Parity		
Parity 1	543	28.98
Parity 2	464	24.76
Parity 3	329	17.56
Parity 4	223	11.9
Parity 5+	315	16.81
Assisted delivery		
No skilled person	583	31.11
Skilled person	1291	68.89
ANC attendance during last pregnancy		
Yes	1841	98.29
No	32	1.71
Is this your first live birth		
Yes	543	28.98
No	1331	71.02

n = 1873

*SD* standard deviation

^a^Number of respondents across some variables categories may not add up to 1873 due to missing data

### Crude model

Table 2 shows both the crude and adjusted models of determinants of SBA at delivery. The crude model revealed that maternal age is significantly associated with SBA at delivery. Participants aged 20–24, 25–29, 30–34 and 40+ were 34, 87, 74 and 45% more likely to be delivered by skilled person respectively compared with those aged 15–19 years (OR 1.34, 95% CI 0.97–1.85), (OR 1.87, 95% CI 1.34–2.60), (OR 1.74, 95% CI 1.22–2.48), (OR 1.45, 95% CI 0.89–2.36). While the odds of having SBA at delivery increased significantly by 69% for women who were married compared to those who were single (OR 1.69, 95% CI 1.25–2.29), women who were separated/divorced, cohabiting and widowed had an increased odds but it was not statistically significant. Level of education had significant influence on SBA at delivery such that, women who had primary education were 61% more likely to have SBA at delivery compared to those who had no education (OR 1.61, 95% CI 1.26–2.05) and those who had junior high/middle school level of education were three times more likely to have SBA at delivery (OR 3.09, 95% CI 2.40–4.00) and those who had senior high and above level of education were more than eight times more likely to have SBA at delivery (OR 8.88, 95% CI 4.90–16.10) compared to those who had no education. While the odds of having SBA at delivery for women who were farmers significantly reduced by 63% compared to those who were unemployed (OR 0.63, 95% CI 0.47–0.85), women who were petty traders had a significant increased odds of 43% of having SBA at delivery compared to those who were unemployed (OR 1.43, 95% CI 1.09–1.88). Women who were artisans and civil servants were two and seven times more like to have SBA at delivery respectively (OR 2.30, 95% CI 1.59–3.33), (OR 7.20, 95% CI 1.69–30.60) compared to those who had no employment.

**Table 2:** Crude and adjusted odd ratios of determinants of skilled birth attendants at delivery

Characteristics	Crude		Adjusted	
	OR	P values (95% CI)	OR	P values (95% CI)*
Age group				
15–19	1		1	
20–24	1.34	0.079 (0.97–1.85)	1.53	0.039 (1.02–2.30)
25–29	1.87	<0.001 (1.34–2.60)^†^	2.12	0.002 (1.31–3.42)^†^
30–34	1.74	0.002 (1.22–2.48)^†^	2.44	0.002 (1.40–4.24)^†^
35–39	2.15	<0.001 (1.42–3.25)^†^	4	<0.001 (2.10–7.61)^†^
40+	1.45	0.139 (0.89–2.36)	4.25	<0.001 (2.08–8.71)^†^
Marital status				
Single	1		1	
Married	1.69	<0.001 (1.25–2.29)^†^	1.32	0.192 (0.87–1.10)
Separated/divorced	1.56	0.289 (0.687–3.54)	3.4	0.036 (1.08–10.68)^†^
Cohabiting	1.09	0.460 (0.87–1.36)	1.2	0.246 (0.88–1.64)
Widowed	1.82	0.459 (0.37–8.836)	2.19	0.490 (0.24–20.38)
Missing	0.88	0.760 (0.40–1.97)	0.97	0.956 (0.39–2.42)
Level of education				
No education	1			
Primary	1.61	<0.001 (1.26–2.05)^†^	1.52	0.006 (1.13–2.06)^†^
Junior high/middle school	3.09	<0.001 (2.40–4.00)^†^	2.07	<0.001 (1.51–2.83)^†^
Senior high and above	8.88	<0.001 (4.90–16.10)^†^	3.41	0.001 (1.65–7.04)^†^
Occupation				
Unemployed	1			
Farmer	0.63	0.003 (0.47–0.85)^†^	0.69	0.050 (0.48–1.00)
Artisan	2.3	<0.001 (1.59–3.33)^†^	1.04	0.856 (0.67–1.63)
Trader	1.43	0.010 (1.09–1.88)^†^	1.25	0.200 (0.89–1.75)
Civil servant	7.2	0.007 (1.69–30.60)^†^		
Student	0.95	0.756 (0.69–1.30)	0.87	0.533 (0.56–1.34)
Others	1.4	0.355 (0.69–2.87)	1.25	0.609 (0.53–2.91)
Parity				
Parity 1	1		1	
Parity 2	0.74	0.029 (0.56–0.97)^†^	0.46	<0.001 (0.32–0.66)^†^
Parity 3	0.76	0.075 (0.56–1.03)	0.34	<0.001 (0.22–0.52)^†^
Parity 4	0.84	0.331 (0.59–1.19)	0.5	0.008 (0.30–0.83)^†^
Parity 5+	0.41	<0.001 (0.30–0.55)^†^	0.24	<0.001 (0.14–0.39)^†^
Socio economic status				
Poorest	1		1	
Poorer	1.39	0.034 (1.03–1.88)^†^	1.17	0.342 (0.84–1.63)
Poor	1.7	0.001 (1.23–2.34)^†^	1.31	0.129 (0.92–1.86)
Less poor	3.54	<0.001 (2.53–4.96)^†^	2.55	<0.001 (1.77–3.69)^†^
Least poor	8.75	<0.001 (5.60–13.67)^†^	4.8	<0.001 (2.96–7.77)^†^
ANC attendance				
Attend ANC	1		1	
Don’t attend ANC	0.03	<0.001 (0.01–0.12)^†^	0.05	<0.001 (0.01–0.22)^†^
Type of live birth				
First live birth	1		1	
Not first live birth	0.65	<0.001 (0.52–0.82)^†^		

*OR* odd ratio, *CI* confidence interval

^†^Statistically significant

* Correct classification rate of the model = 72.65%

Parity has shown significant effect on use of SBA at delivery. Parity 2 and 5 had significantly increased odds of 74 and 41% respectively compared to those with parity 1 (OR 0.74, 95% CI 0.56–0.97), (OR 0.41, 95% CI 0.30–0.55). The rest of the categories of the parity (three and four) were not significantly different (P > 0.05). Socioeconomic status was another variable which strongly predicted having SBA at delivery. While women with socioeconomic status of poorer and poor had significantly increased odds of 39 and 70% of having SBA at delivery respectively compared to those in the poorest category (OR 1.39, 95% CI 1.03–1.88), (OR 1.70, 95% CI 1.23–2.34), less poor and least poor women were more than three and eight times more likely respectively to have SBA at delivery compared to those who were poorest (OR 3.54, 95% CI 2.53–4.96), (OR 8.75, 95% CI 5.60–13.67). The results further showed that, women who do not attend ANC were 3% less like to have SBA at delivery compared to their counterparts who attend ANC (OR 0.03, 95% CI 0.01–0.12). Finally, women who ever had live birth were 65% less likely to have SBA at delivery compared to those who never had live birth (OR 0.65, 95% CI 0.52–0.82).

### Adjusted model

The adjusted model assessed the independent effect of each determining variable on SBA at delivery. The model contain eight determinates (age group, marital status, level of education, occupational status, parity, socioeconomic status, ANC attendance and type of birth) of SBA at delivery with over all correct classification rate of 72.65%. The result shows a remarkable influence of maternal age, level of education, parity, socioeconomic status and ANC attendance on SBA at delivery.

The odds of women aged 20–24 and 25–29 years having SBA at delivery is 2.12 and 2.44 times more likely compared to those aged 15–19 years respectively (OR 2.12, 95% CI 1.31–3.42), (OR 2.44, 95% CI 1.40–4.24). Women aged 35–39 and 40+ years were significantly four times and more than four times more likely to have SBA at delivery respectively compared to those aged 15–19 years (OR 4.00, 95% CI 2.10–7.61), (OR 4.25, 95% CI 2.08–8.71).

With respect to level of education, while women with primary education were 52% more likely to have SBA at delivery (OR 1.52, 95% CI 1.13–2.06), those with junior high/middle school education were more than two times more likely to have SBA at delivery compared to those with no education (OR 2.07, 95% CI 1.51–2.83). Women with senior high and above level of education were three times more likely to have SBA at delivery compared to those in the reference category. Parity was another variable that exerted significant influence on SBA at delivery. While women with parity 2 were 46% less likely to have SBA at delivery (OR 0.46, 95% CI 0.32–0.66), those with parity 3 were 34% less likely to have SBA delivery compared to those with parity of 1 (OR 0.34, 95% CI 0.22–0.52). Women with parity 4 and 5 had a reduced odds of 0.50 and 0.24 respectively compared to those with parity 1 (OR 0.50, 95% CI 0.30–0.83), (OR 0.24, 95% CI 0.14–0.39).

The results showed further that women in less poor socioeconomic quintile were more than twice more likely to have SBA at delivery (OR 2.55, 95% CI 1.77–3.69) and those in least poor quintile were more than four times more likely to have SBA at delivery compared to those in the poorest quintile (OR 4.80, 95% CI 2.96–7.77). Although other socioeconomic quintiles were not significantly associated with SBA at delivery (P > 0.05), the odds of SBA at delivery was increasing with improvement in socioeconomic status. Furthermore, women who do not attend ANC were 5% less likely to have SBA at delivery compared to those who attend ANC (OR 0.05, 95% CI 0.01–0.22).

## Discussions

This study sought to explore determinants of skilled birth attendants at delivery using data from Dodowa Health and Demographic Surveillance System. Data was collected from Shai-Osudoku and Ningo-Prampram districts of southern rural Ghana in 2011. The results show that SBA at delivery was high in the study area, with 68.89% coverage. This proportion is higher than the national estimate of 52.2%, Greater Accra regional estimate of 56.0% from 2011 Ghana Health Services annual report [31] and DWD estimate of 37.0% for the same year [32]. Our finding is similar to the national estimates of 68% reported by Ghana Statistical Services in 2011 Ghana Multiple Indicator Cluster Survey (MICS) [27].

The result shows that socioeconomic status is a significant determinant of SBA at delivery in that, better socioeconomic status was significantly associated with SBA at delivery. This implies that the user-fee exemption policy for maternal and child health services may have had less impact on the use of SBA because there are other costs including transport, hence reducing user-fees alone may not be sufficient motivation to bring in the poorest women.

This finding is consistent with a qualitative study in Ghana [33], 2011 Ghana MICS report [27] and other studies elsewhere [34, 35] where women from poorest households were less likely to deliver using skilled personnel compared to women from the richest households.

Furthermore, the result on level of education and SBA at delivery is consistent with the finding of 2011 Ghana MICS report which revealed that the more educated a women is, the more likely she is to have SBA at delivery [27]. This finding is further supported by other studies [36, 37]. Educated women may have better jobs and higher socioeconomic status which also enhances their negotiation power and confidence in making informed decision on place of delivery and SBA utilization [36, 38].

The finding on parity and SBA utilization is consistent with similar studies elsewhere [36, 39, 40]. Women with higher parity have experienced childbirth and takes childbirth as a natural process hence may not seek SBA at delivery if there is no complication [39]. It has also been shown that, if a woman had a bad experience with a health worker during previous childbirth, she may prefer delivering subsequent babies at home instead [36].

It has been established that ANC is the first step of continuum of services provided during pregnancy [41] and mothers who attends ANC are likely to attend health facility for childbirth [36, 42, 43]. This study revealed significant positive association between ANC attendance and SBA at delivery such that women who do not attend ANC were less likely to have SBA at delivery. Several other studies also found positive association between ANC attendance and facility delivery [42–44].

Finally, the result from this study shows older women were more likely to have SBA at delivery. Other similar studies established that advance maternal age is a risk factor for a woman to undergo induction and caesarean delivery [45–47] hence older women were encouraged to deliver in health facility so as to have SBA at delivery.

The major strength of this study is use of data from Health and Demographic Surveillance System which follows the entire population of the study area prospectively [29] hence the generalizability of the finding to the whole population.

## Conclusion

Although 69% of women in the study area had skilled birth attendants at delivery, women from poorest households, higher parity, uneducated, and not attending ANC and younger women were more likely to deliver without a skilled birth attendants at delivery.

Most of the factors that predicted having a SBA in this rural area of Ghana have also been identified in previous studies [27, 33–47]. Future intervention in the study area to bridge the gap between the poor and least poor women, improve maternal health and promote the use of skilled birth at delivery is recommended.

## Abbreviations

ANC: antenatal care
CHPS: Community-based Health Planning and Services
DHDSS: Dodowa Health and Demographic Surveillance System
DHRC: Dodowa Health Research Centre
EOC: emergency obstetric care
GDHS: Ghana Demographic Health Survey
MDG: Millennium Development Goal
PCA: principal component analysis
SBA: skilled birth attendant
OR: odd ratio
